# An uncomplicated method for growing nano-quasicrystalline structures in the AlCuFeB quaternary alloy system: A short-time milling

**DOI:** 10.1016/j.mex.2021.101305

**Published:** 2021-03-17

**Authors:** Meysam Amini, Mohammad Reza Rahimipour, Seyed Ali Tayebifard, Yahya Palizdar

**Affiliations:** a Ceramic Department, Materials and Energy Research Center, Karaj, Iran; b Semiconductor Department, Materials and Energy Research Center, Karaj, Iran; c Nanotechnology and Advanced Materials Department, Materials and Energy Research Center, Karaj, Iran

**Keywords:** Nanoquasicrystal, Mechanical alloying, Spectral absorption

## Abstract

Quasicrystals have been comprehensively studied since their discovery in 1984 by Daniel Shechtman to dissolve their complex structures concerning their physical properties [1], [2], [3], [4], [5]. The quasi-periodic well-ordered atomic structures made QCs as a novel type of solids differing from the crystalline or amorphous materials [5], [6], [7]. The QC alloys have non-crystallographic rotational symmetries, such as five-fold, eight-fold, and even ten-fold rotational axes that are prohibited in ordinary periodic crystals [5], [6], [7]. The QC alloys have been recently considered for several applications owing to a series of the unusual combination of exclusive physicomechanical properties for metallic-based alloys [5], [6], [7]. Accordingly, the properties of these alloys can be pointed out to their high strength, elevated hardness, desirable corrosion and wear resistance, minute adhesion, low values of thermal and electrical conductivity, and superior optical properties [5], [6], [7].

• This paper focuses on the synthesis, structural and microstructural evolutions, thermal stability, microhardness, and electrical and optical properties of the Al_59_Cu_25.5_Fe_12.5_B_3_ nanoquasicrystalline alloy for solar selective absorber usages. Accordingly, there are various advanced techniques for synthesizing the nanostructured quasicrystals, such as laser ablation, sol-gel, electros pining, mechanical alloying, and hydrothermal methods.
• The structural and microstructural evolutions of the mechanically alloyed AlCuFeB powders were investigated by X-ray diffractometry and field-emission scanning electron microscopy. The thermal stability of the AlCuFeB powders was recorded by differential thermal analysis.
• The nanostructured Al_59_Cu_25.5_Fe_12.5_B_3_ stable quasicrystalline phase was synthesized by short-time milling procedure in 1 h. It was found that the presence of the quasicrystalline phase in the AlCuFeB alloy prominently improves the microhardness, electrical resistivity, and sunlight absorptance.

## Graphical abstract

**Figure:**
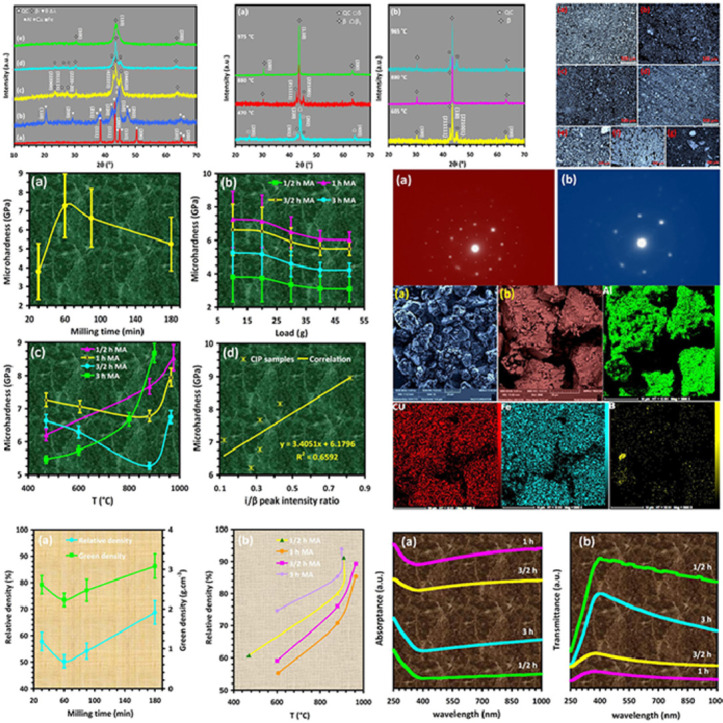

Specifications table

**Table:** 

Subject Area:	Materials science
More specific subject area:	*Nanostructured materials, Quasicrystals, Characterization*
Method name:	*Mechanical alloying*
Name and reference of original method:	*C. Suryanarayana, Mechanical alloying and milling, Prog. Mater. Sci. 46 (2001) 1–184.*https://doi.org/10.1016/S0079–6425*(99)00010-9.*
Resource availability:	https://iopscience.iop.org/article/10.1088/2053–1591/ab9b37/meta *M. Amini, M.R. Rahimipour, S.A. Tayebifard, Y. Palizdar, Effect of milling time on XRD phases and microstructure of a novel Al 67 Cu 20 Fe 10 B 3 quasicrystalline alloy, Mater. Res. Express 7 (2020) 065011.*https://doi.org/10.1088/2053–1591/ab9b37*. https://www.sciencedirect.com/science/article/abs/pii/S0921883120304325M. Amini, M.R. Rahimipour, S.A. Tayebifard, Y. Palizdar, Ultrafast synthesis of the nanostructured Al59Cu25. 5Fe12. 5B3 quasicrystalline and crystalline phases by high-energy ball milling: Microhardness, electrical resistivity, and solar cell absorptance studies, Adv. Powder Technol. 31 (2020) 4319–4335.https://doi.org/10.1016/j.apt.2020.09.013* https://www.sciencedirect.com/science/article/abs/pii/S0925838820342353 *R. Rawat, A. Tiwari, N. Arun, S. V. S. Nageswara Rao, A. P. Pathak, Y. Shadangi, N. K. Mukhopadhyay, S. Venugopal Rao, A. Tripathi, Nanosecond pulsed laser ablation of Al–Cu–Fe quasicrystalline material: Effects of solvent and fluence, J. Alloys Compd. (2021) 157871.* https://doi.org/10.1016/j.jallcom.2020.157871.

## Method details

The MA process is unique in the sense that it saves a lot of time and energy for the synthesis of the QC phase. The nanoquasicrystalline phase commonly takes more than 40–50 h to synthesize by high-energy ball milling at 200 rpm. However, in this article, an attempt has been made to the fast synthesis of the QC phase in the AlCuFeB alloy system through short-time mechanical alloying at 600 rpm. In this work, the AlCuFeB alloy compounds were synthesized by constituent elements in a mechanical milling device. The high purity elemental powders of aluminum (99%), copper (99.7%), iron (99.5%), and boron (99.9%) with an average particle size of less than 200 µm corresponding to the nominal composition of the Al_59_Cu_25.5_Fe_12.5_B_3_ (at.%) alloy system were utilized as the starting reactant materials to be mechanically alloyed. The milling process occurred in a Retsch PM 400 high-energy planetary ball mill machine, and the consolidation procedure of the as-milled powders was conducted using a KJYc300 CIP apparatus under a pressure of 400 MPa at room temperature with a soaking time of 5 min. The as-milled powders were loaded into a cylindrical elastomeric mold to fabricate condensed specimens of 30 mm in diameter with maximum green density, based on the compressibility of the as-milled powders. The MA process was accomplished at a rotation speed of 600 rpm under a high purified dry argon atmosphere to diminish oxygen and nitrogen pollution. The individual high-grade elemental powders were mixed in a glove box under a controlled atmosphere (high purified argon) to obtain the demanded stable QC phase besides related crystalline intermetallics using appropriate stoichiometric proportions to prepare the accurate nominal composition of the Al_59_Cu_25.5_Fe_12.5_B_3_ alloy system. The milling procedure was carried out employing the hardened steel balls and vials at a ball to powder weight ratio of 45 to 1. The mixed elemental powder batch was charged into a 500ml-volume hardened steel vial The milling vials maintained a powder batch of 10 g total weight accompanying with thirteen 20 mm (in diameter) balls as the milling media. An organic surfactant (1 wt% stearic acid) is appended to the vials during milling progression as a process control agent (PCA) to increase the performance of the free-running ball-milled CMA-powders. Additionally, the aforementioned organic surfactant reduces the adhesion effect of the soft species into the milling medium (balls and vials wall surface). It also prevents surplus cold welding of the raw materials. The milling process was periodically discontinued after each 1/2 h to bypass an increase in the vial temperature and overheating the powder blend as well as cooling the milling apparatus. Afterwards, the rotation direction was switched, and the milling process was started again. The milling process time was engaged to be 1/2 to 3 h since the formation of the QC phase, and its transformation into the β-Al (Cu, Fe) crystalline phase occurred in this period time only with high milling speed at 600 rpm. The vials were opened at particular milling times in an isolated glove box, and about 1 g of the ball-milled powders was removed for further operations and characterizations. The effect of the heat treatment process on the formation of the QC phases and other intermetallic compounds in the Al_59_Cu_25.5_Fe_12.5_B_3_ was investigated. Accordingly, succeeding isothermal annealing procedure was performed on the as-milled powders under the flowing of the argon atmosphere at predetermined temperatures for 4 h [7].

The structural evolutions and characterization of the produced phases in each specimen were identified by X-ray diffraction (XRD). It was examined using a PHILIPS-PW 3710 multipurpose diffractometer with Cu Kα radiation (λ_Cu-Kα_=1.54 Å) performing at 40 kV tube voltage and 30 mA current in the Bragg angle range of 20°−70° with step time of 0.6 s and step size of 0.01. The main reason for choosing the XRD angle between 20 and 70° and not prolonging to 90° is that the most XRD peaks of different phases are located in this range of XRD angle. The XRD patterns were scrutinized utilizing PANalytical HighScore and MAUD software. The microstructure, morphology, elemental distribution, chemical microanalysis, and particle size of desired samples were assessed using a TESCAN-MIRA3 field-emission scanning electron microscope (FESEM) equipped with energy-dispersive X-ray spectroscopy (EDS) operating at 5 kV. The EDS examination was carried out, including both point and map modes at the identical operating voltage. The electron diffraction pattern of the 1 h milled powders containing QC i-phase was performed by a Tecni G high-resolution transmission electron microscope (HRTEM) at 200 kV operating voltage. Moreover, the microstructure of the bulk specimens produced in the CIP procedure was investigated by the Olympus PME3 optical microscope (OM) and recorded employing the Axio Vision image analysis. The particle size distribution of the as-milled AlCuFeB powders was measured using the static light scattering (SLS) method by a Fritsch Particle Size Analysette 22 (Laser Particle Sizer). The approximate composition of the as-milled AlCuFeB powders (boron and other elements contents) was examined using a PerkinElmer SCIEX ELAN DRC-2 inductively coupled plasma-atomic emission spectroscopy (ICP-AES). The thermal stability of the powders was recorded by differential thermal analysis (DTA). The weight gain of the particles during the heat treatment was investigated through thermogravimetric analysis (TGA). They were performed concurrently on a BAHR-STA 503 with a constant heating rate of 10 °C/min under a high purified argon atmosphere using a 300cm^3^/min flow rate. The created phases in any samples throughout the DTA examinations were characterized by the XRD investigation. The Vickers microhardness of the consolidated as-milled and heat-treated samples was measured using a computer-controlled Akashi MVK-H1 device equipped with a digital video measuring system to confirm that the districts of selected indentation are away from the edges. It must be said that the microhardness measurements were performed at loads of 10 to 50 g and 15 s dwell time. The Vickers microhardness values in each sample were obtained from 10 indentations leastwise. The test results were means of the values, not including the minimum and maximum values. The final green density of each ball-milled powder consolidated by the CIP procedure was assessed via the Archimedes method with de-ionized water as the immersion solution. The electrical resistivity of the consolidated as-milled and heat-treated samples was measured by a standard four-point probe dc method (FPP) at room temperature. The experimental results were delivered based on the means of ten testing samples. The examination results were averages of the values, not involving the minimum and maximum ones. The spectral analyses of absorption in the ultraviolet, visible, and near-infrared (UV–Vis-NIR) areas on the as-milled AlCuFeB consolidated samples fabricated through the CIP technique were carried out using a PerkinElmer Lambda 25 UV/VIS spectrometer operating in the wavelength range of 250–1000 nm. The spectrometer is attached to a fiber-optic reflection probe R200–7-SR located at about 4 mm far from the sample. This setup minimizes the losses related to scattering and causes every diffuse reflectance received at the probe [7].

In the present attempt, the nanostructured Al_59_Cu_25.5_Fe_12.5_B_3_ QC and crystalline particles were synthesized by an optimum MA procedure. The rationale behind using the term short-time synthesis is to synthesize the IQC phase only by the high-energy ball milling procedure in short-term ball milling without subsequent annealing treatment. Moreover, this particular method was used to prevent the possibility of the formation of a single β-phase. Since the β-phase is thermodynamically more stable in comparison to the QC i-phase and under the equilibrium state, it could prevent the formation of the QC i-phase. This work aims to examine the effect of short-time milling on the direct formation possibility of the nanostructured AlCuFeB QC i-phase and related crystalline phases by high-energy planetary BM process. The second part of the work focuses on accurate identification of all phases and their transformations during MA and post-annealing treatments of the Al_59_Cu_25.5_Fe_12.5_B_3_ as-milled powders in more detail. In this paper, we report on an exhaustive study of the microhardness, electrical resistivity, and optical (spectroscopic) properties of the AlCuFeB milled powders. The AlCuFeB milled powders were consolidated with the aid of a cold isostatic pressing (CIP) machine [7].

## Data description

**Fig. 1:**
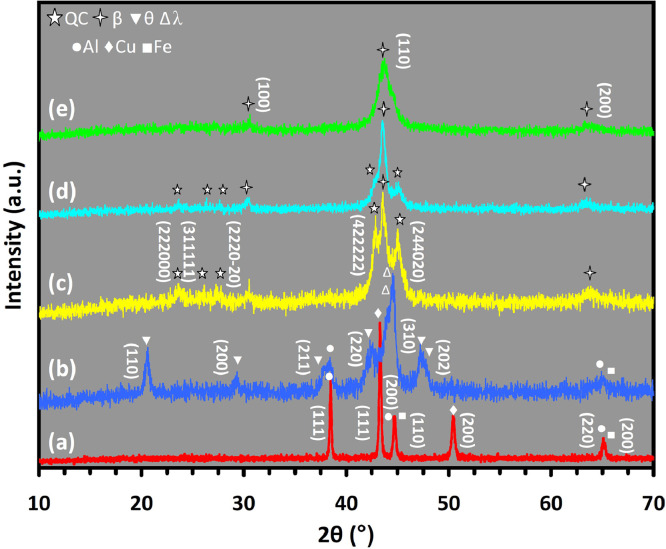
XRD patterns of the mechanically alloyed Al_59_Cu_25.5_Fe_12.5_B_3_ powder particles after (a) 0 h, (b) 1/2 h, (c) 1 h, (d) 3/2 h, and (e) 3 h, signifying the gradual structural evolutions of the θ, λ, β, and QC phases.

**Fig. 2:**
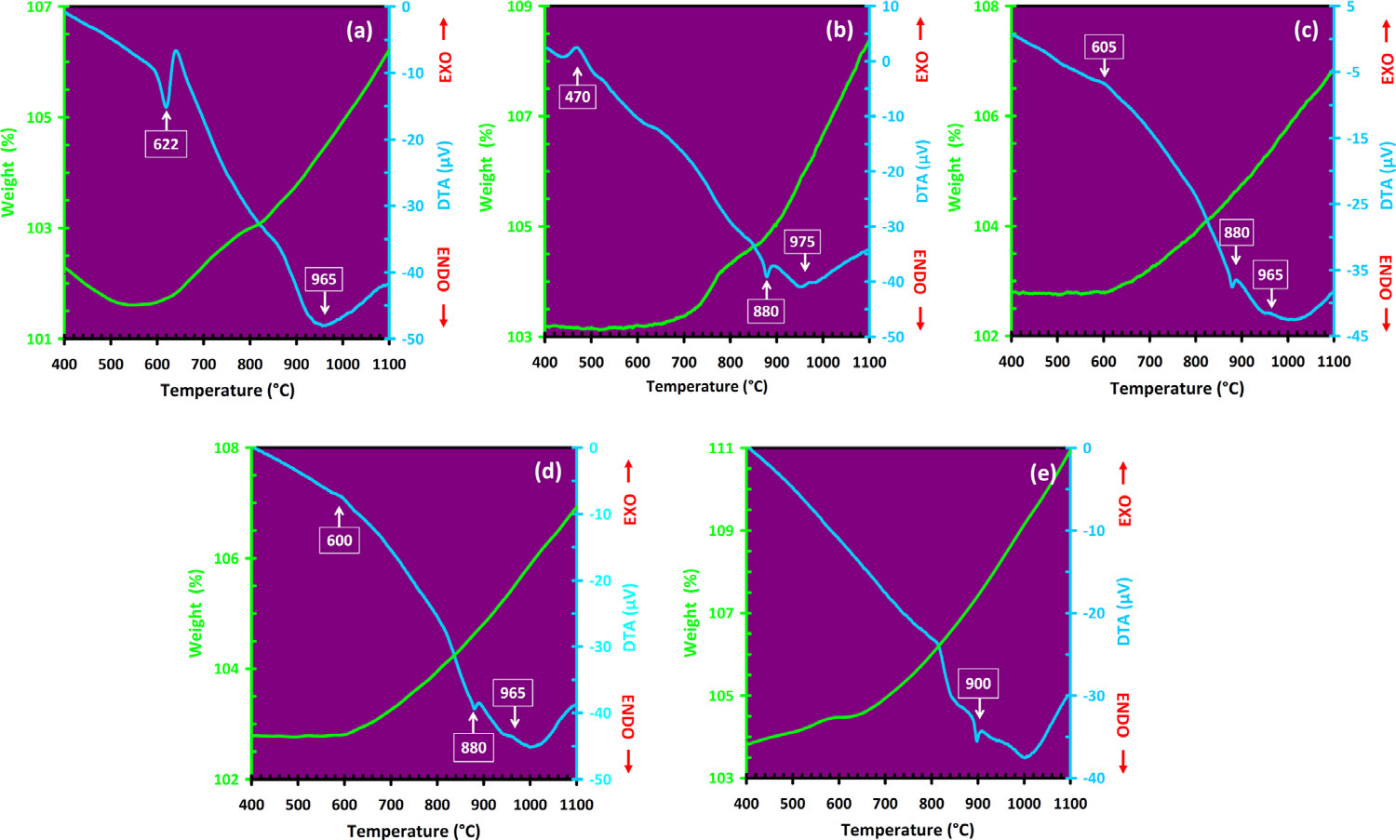
DTA and TGA traces of the (a) un-milled and mechanically alloyed Al_59_Cu_25.5_Fe_12.5_B_3_ powder particles after (b) 1/2 h, (c) 1 h, (d) 3/2 h, and (e) 3 h (e).

**Fig. 3:**
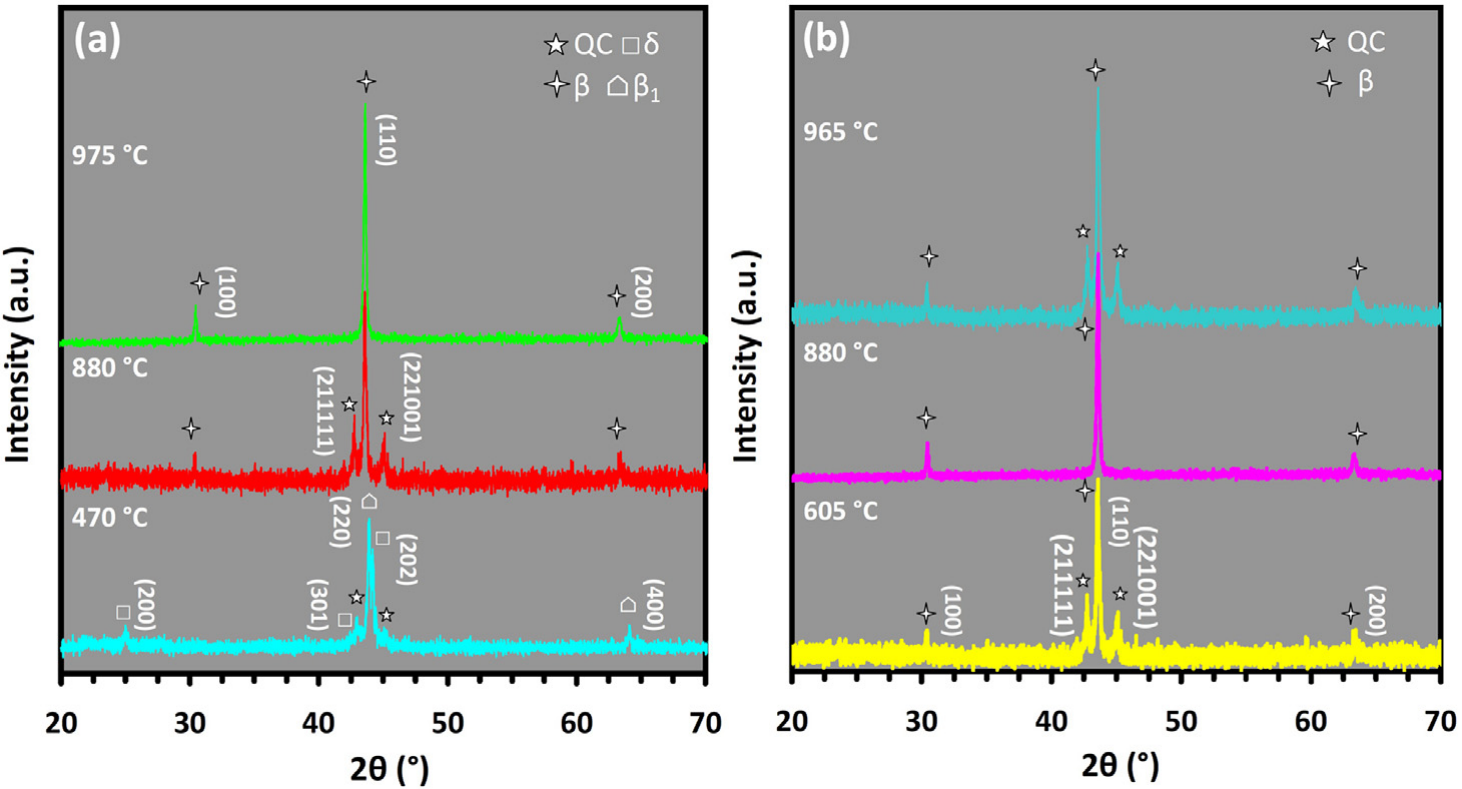
XRD patterns of the heat-treatedAlCuFeB powder particles after (a) 1/2 h and (b) 1 h, presenting the structural evolutions and phase transformations of the as-milled AlCuFeB alloy powders.

**Fig. 4:**
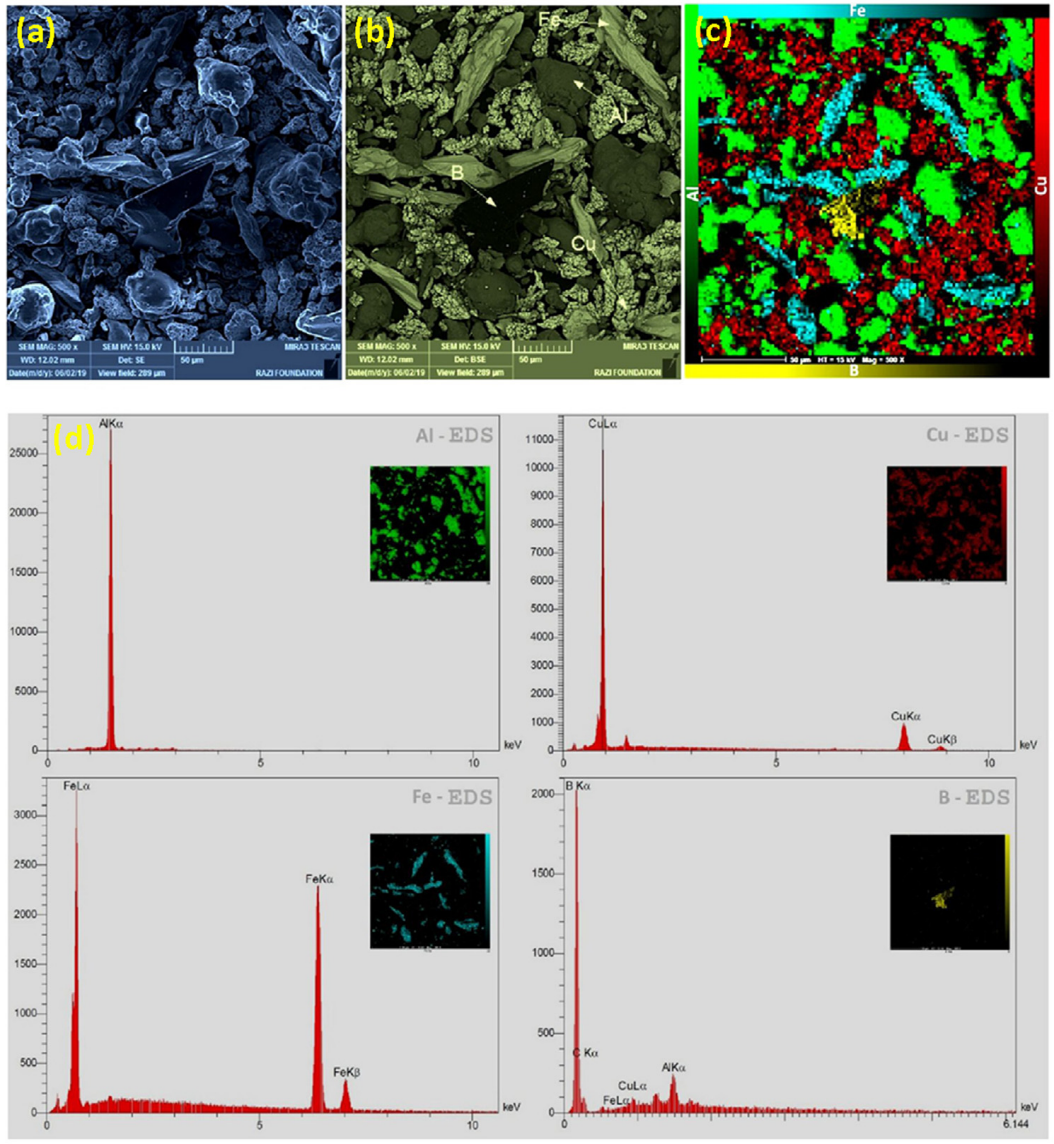
(a) SE-FESEM, (b) BSE-FESEM, (c) map EDS, and (d) corresponding point EDS of the un-milled Al_59_Cu_25.5_Fe_12.5_B_3_ powder particles, showing the size and morphology, microstructure, elemental distribution, and chemical composition of Al, Cu, Fe, and B raw elements.

**Fig. 5:**
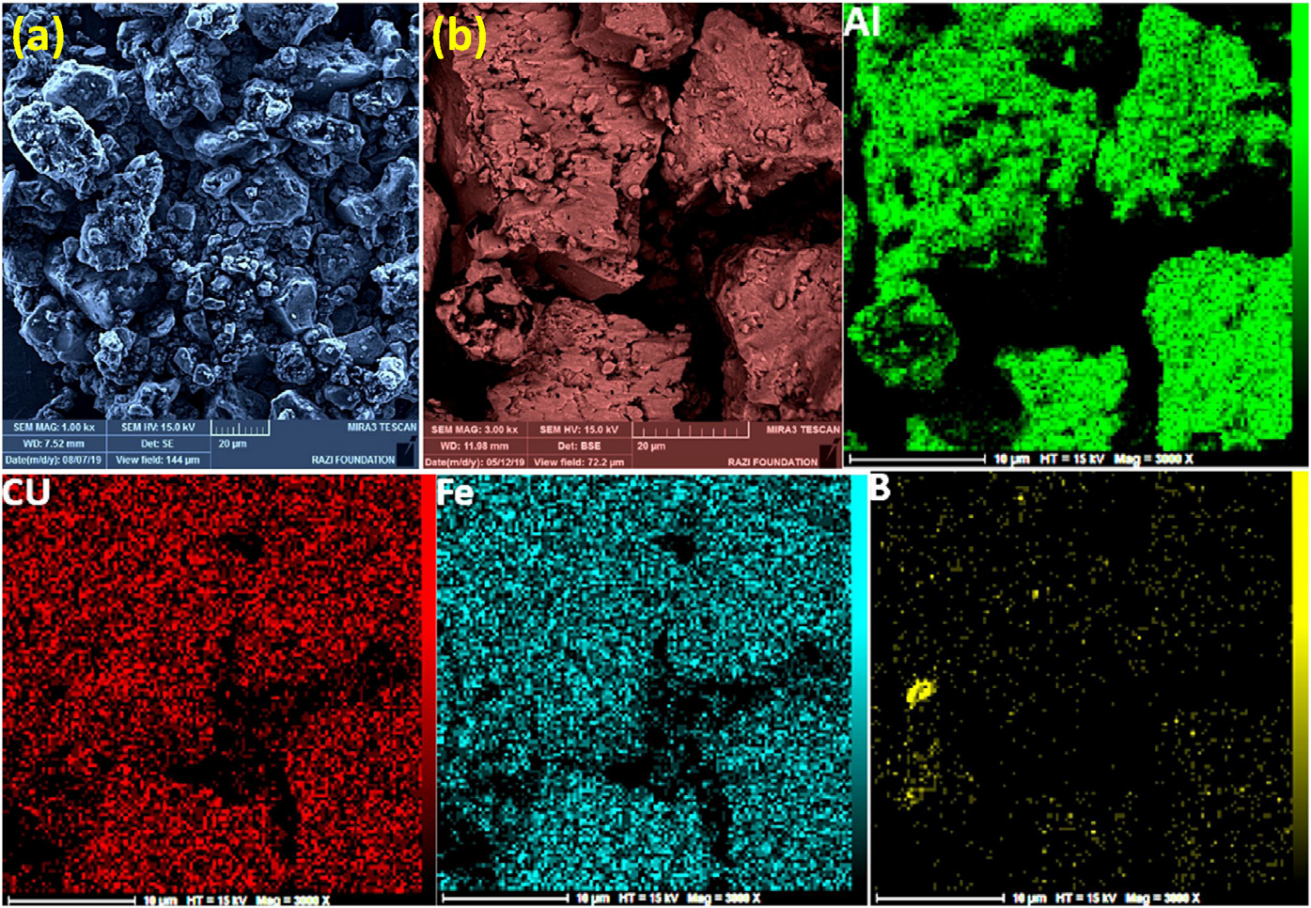
(a) SE-FESEM, and (b) BSE-FESEM images of the 1/2 h mechanically alloyed Al_59_Cu_25.5_Fe_12.5_B_3_ powders beside the EDS mapping corresponded to the BSE image, representing the powder particle size, morphology, microstructure, and elemental distribution of the powder particles.

**Fig. 6:**
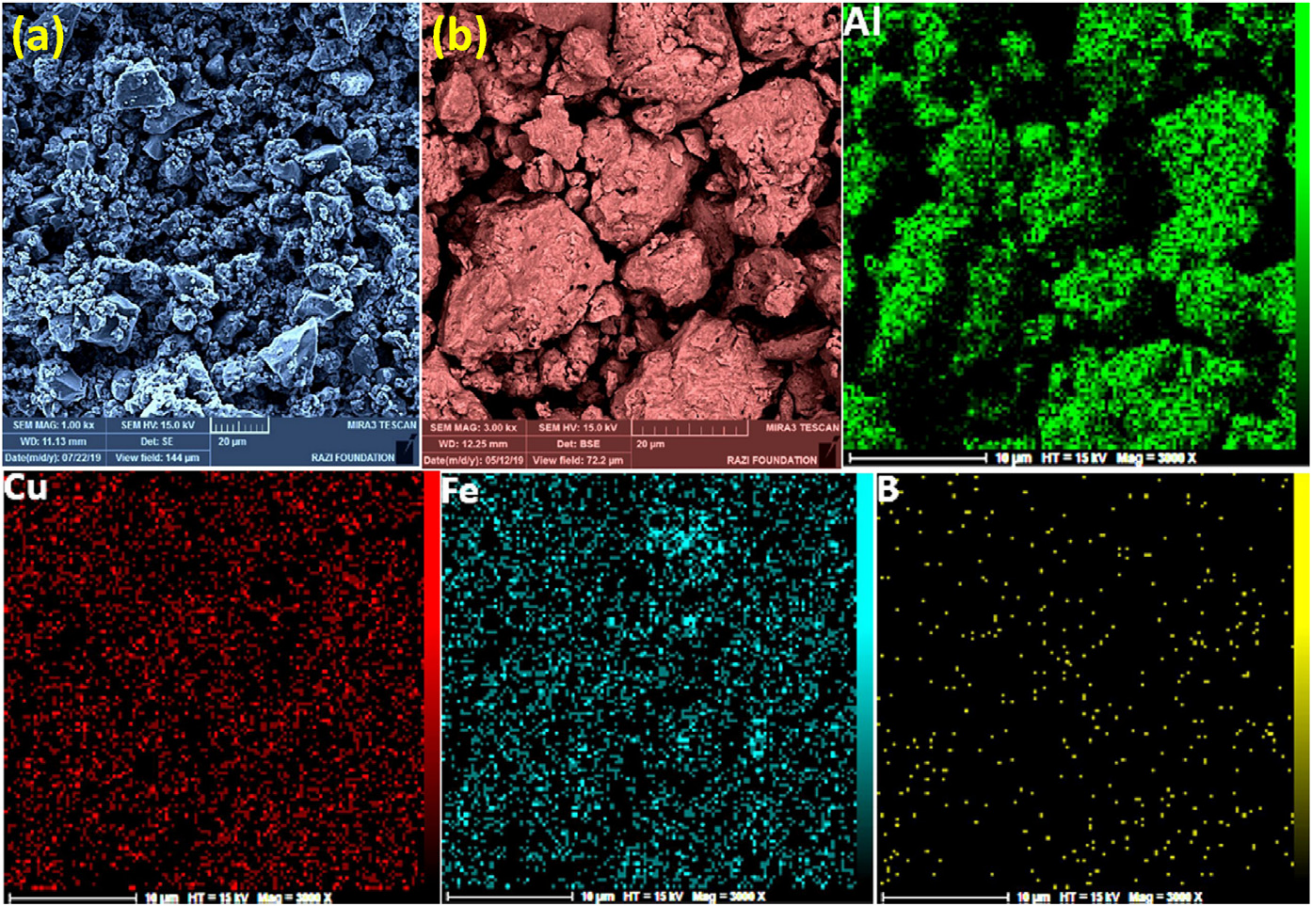
(a) SE-FESEM, and (b) BSE-FESEM images of the 1 h mechanically alloyed Al_59_Cu_25.5_Fe_12.5_B_3_ powders beside the EDS mapping corresponded to the BSE image, describing the powder particle size, morphology, microstructure, and elemental distribution of the powder particles.

**Fig. 7:**
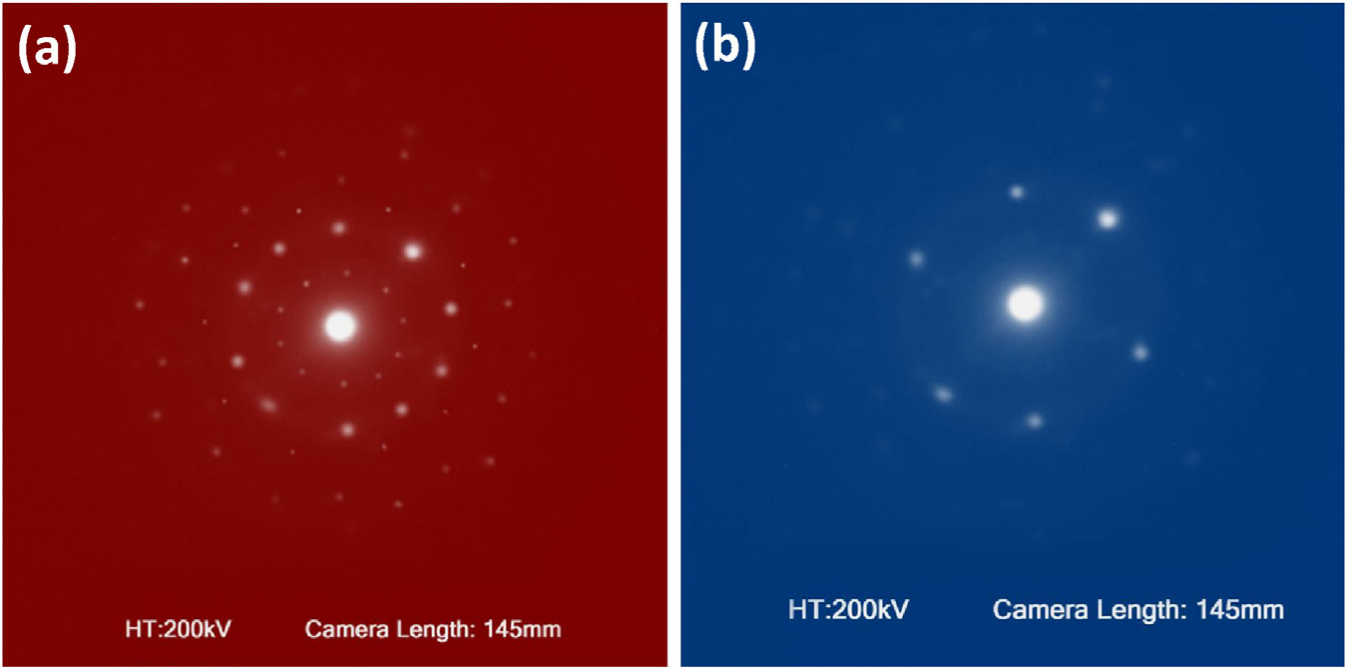
SAD patterns of the Al_59_Cu_25.5_Fe_12.5_B_3_ QC i-phase, showing the presence of five-fold (a), three-fold (b), and two -fold (b) symmetries.

**Fig. 8:**
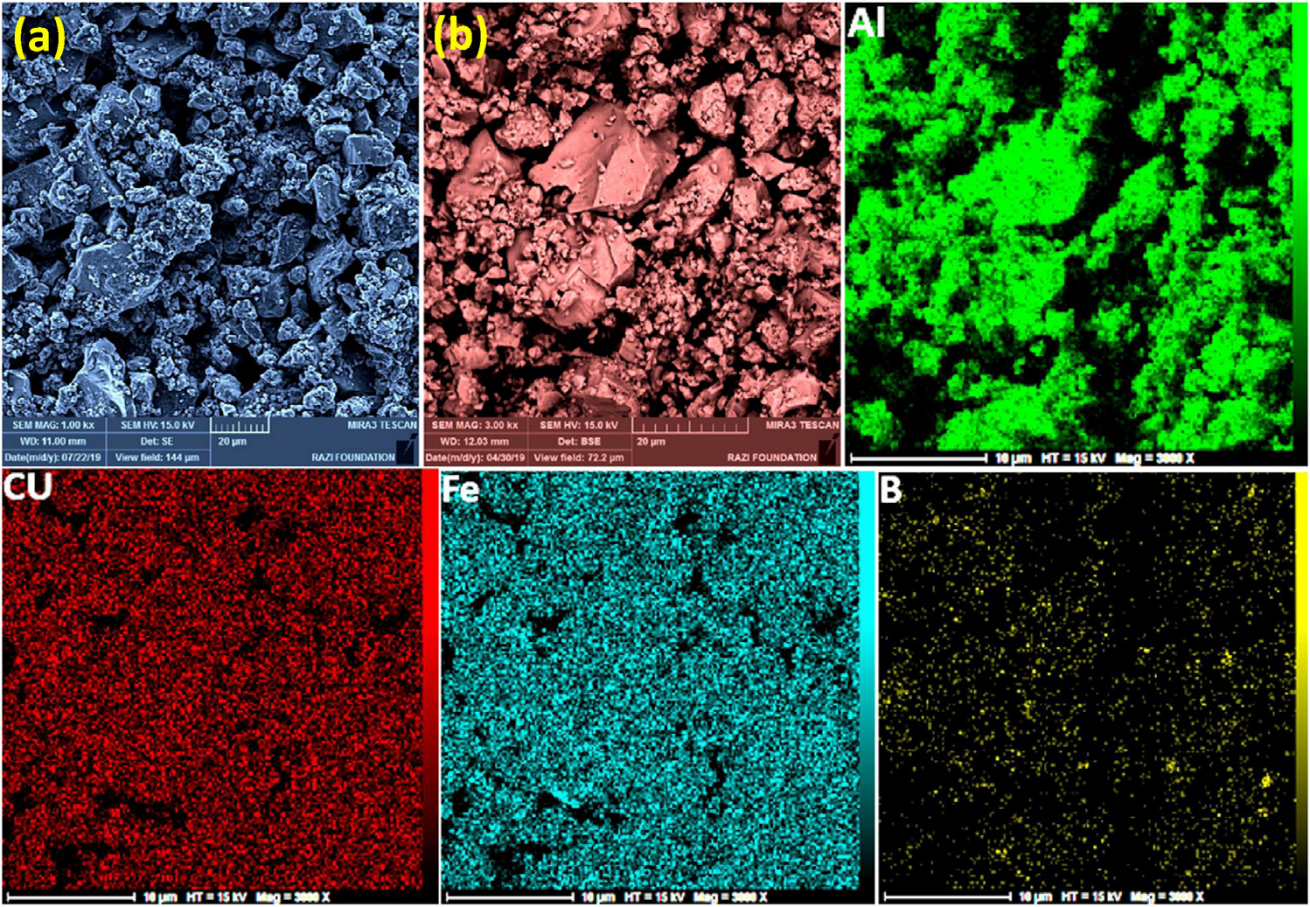
(a) SE-FESEM, and (b) BSE-FESEM images of the 3/2 h mechanically alloyed Al_59_Cu_25.5_Fe_12.5_B_3_ powders beside the EDS mapping corresponded to the BSE image, showing the powder particle size, morphology, microstructure, and elemental distribution of the powder particles.

**Fig. 9:**
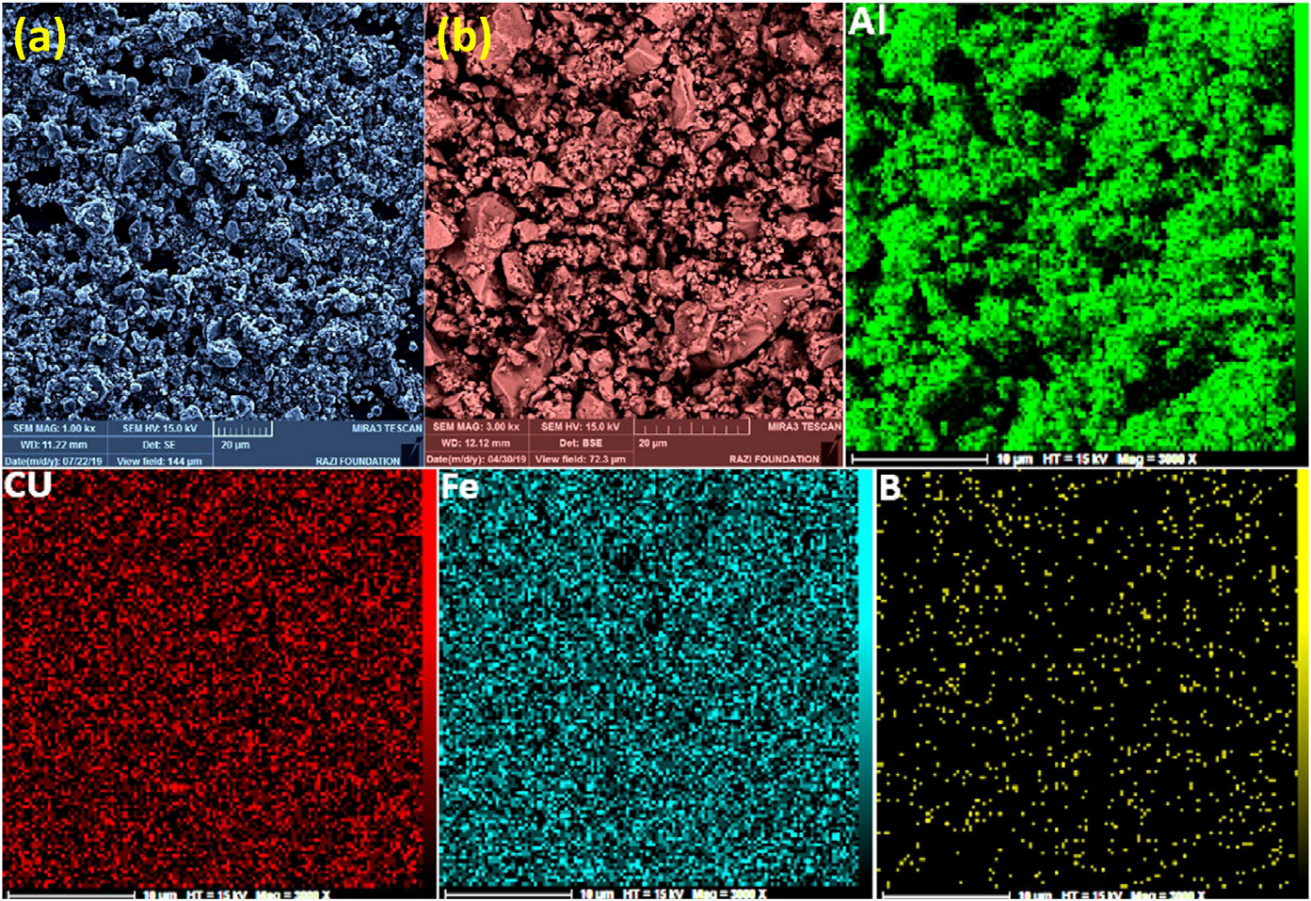
(a) SE-FESEM, and (b) BSE-FESEM images of the 3 h mechanically alloyed Al_59_Cu_25.5_Fe_12.5_B_3_ powders beside the EDS mapping corresponded to the BSE image, presenting the powder particle size, morphology, microstructure, and elemental distribution of the powder particles.

**Fig. 10:**
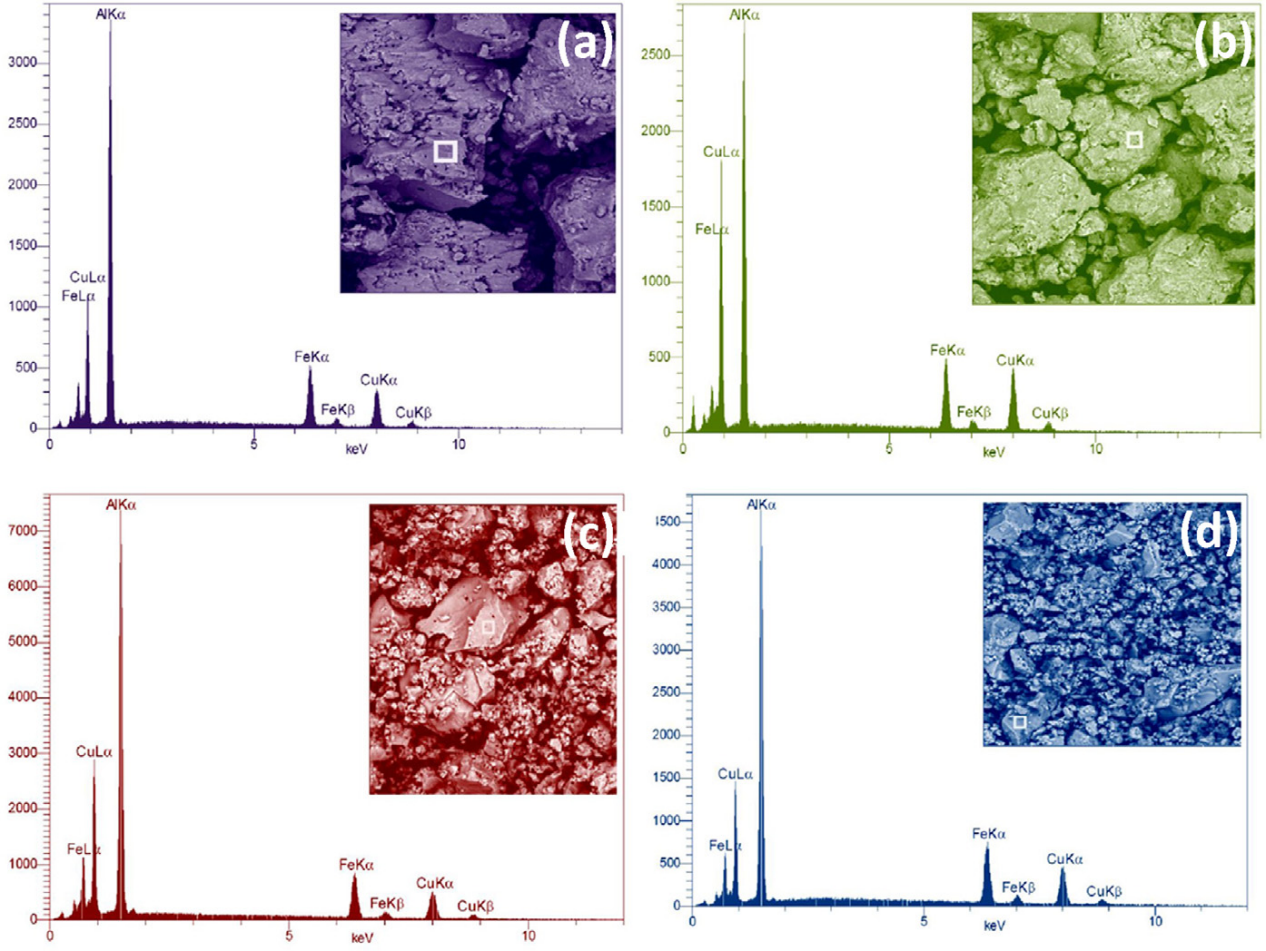
Point EDS of (a) 1/2 h, (b) 1 h, (c) 3/2 h, and (d) 3 h corresponding to the BSE images of the mechanically alloyed Al_59_Cu_25.5_Fe_12.5_B_3_ alloy powders, showing the chemical composition of the selected area of the powder particles.

**Fig. 11:**
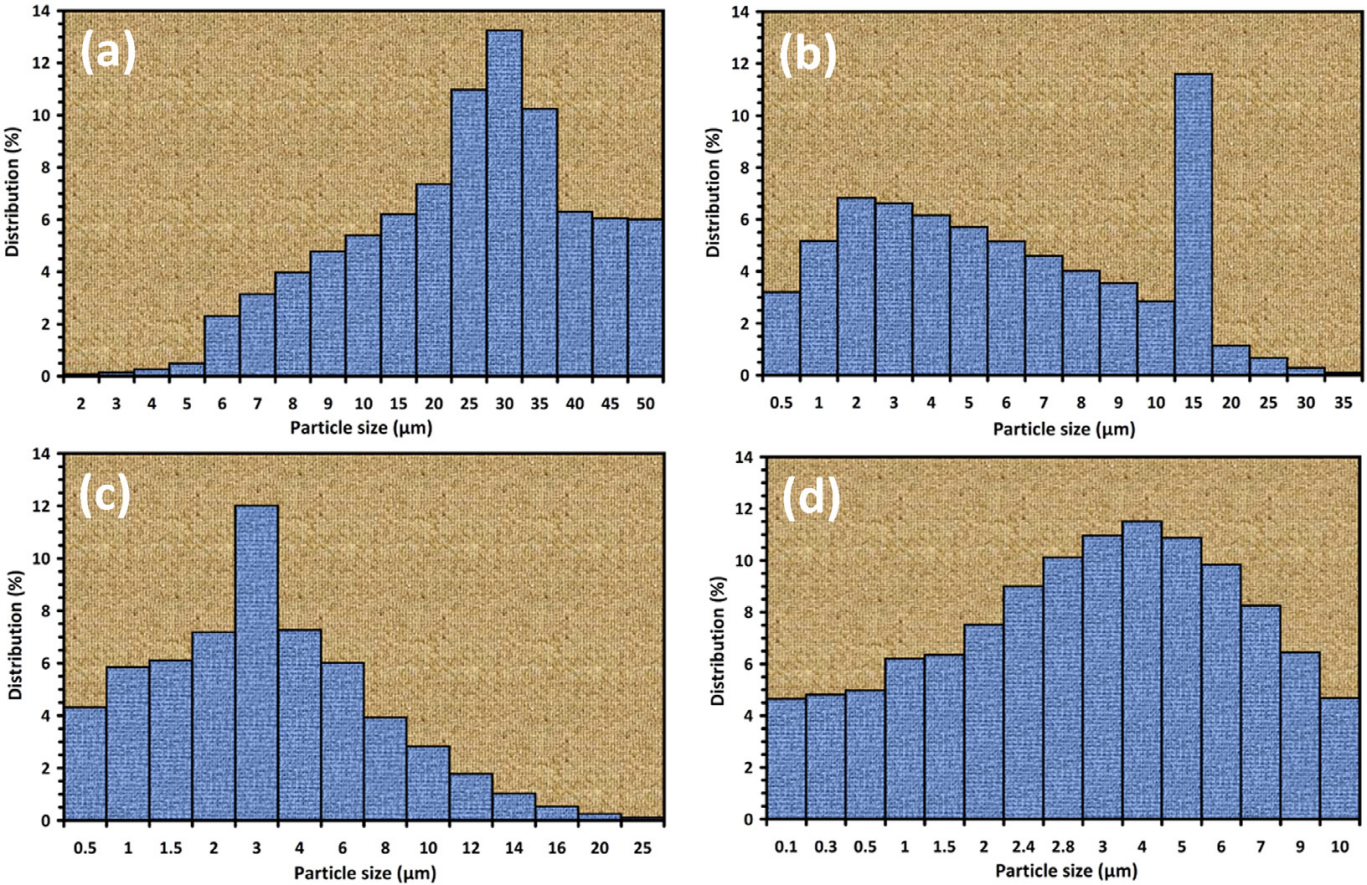
Particle size distribution diagram of the mechanically alloyed AlCuFeB powders: (a) 1/2 h; (b) 1 h; (c) 3/2 h; (d) 3 h. It represents the particle size variations from 0.1 to 50 μm for 1/2 to 3 h MA process and decreasing the average particle diameter of the as-milled powders by the increment of the milling process time.

**Fig. 12:**
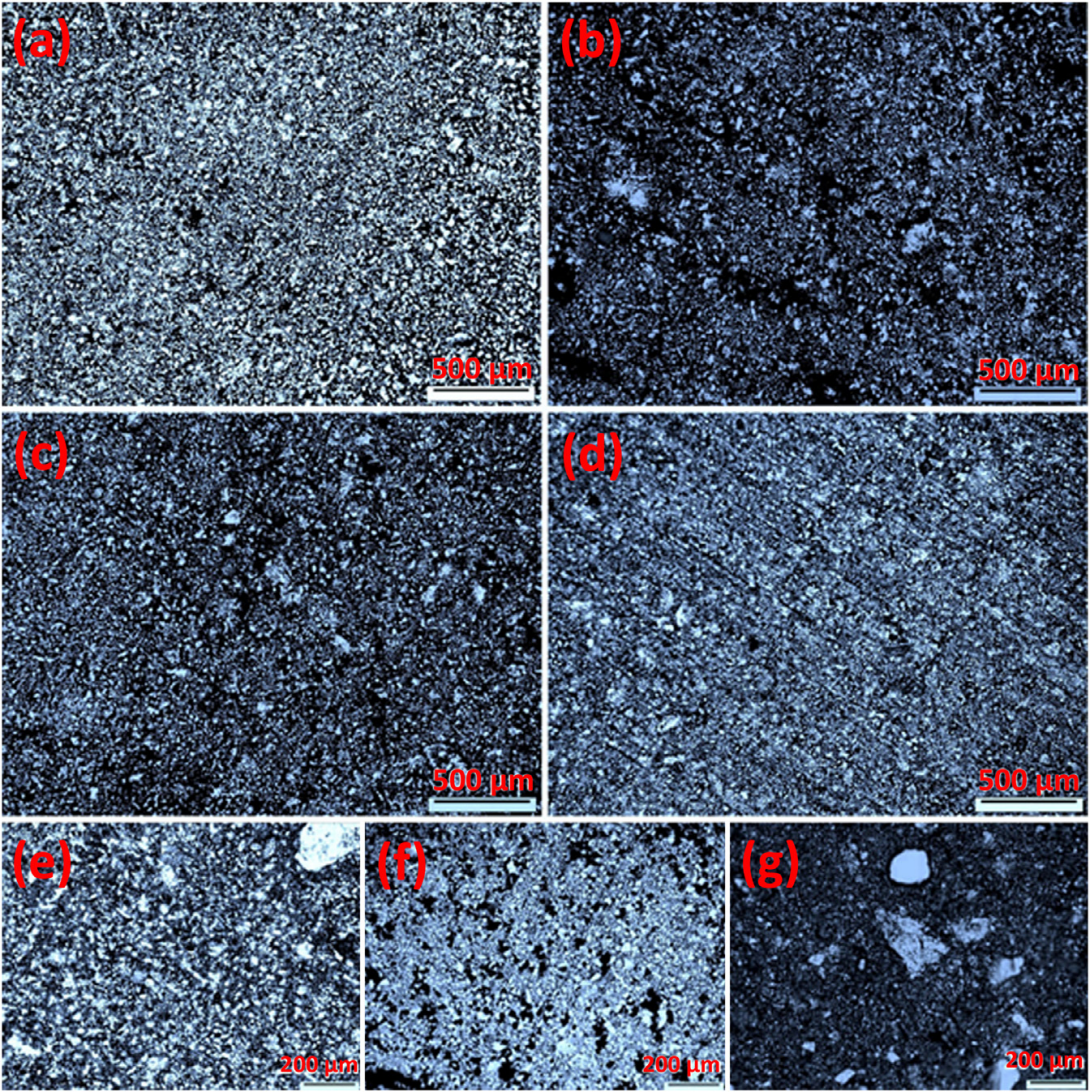
Surface OM images of the consolidated as-milled AlCuFeB alloy powders after (a) 1/2 h, (b) 1 h, (c) 3/2 h, and (d) 3 h, besides, consolidated 1 h ball-milled powders after annealing at (e) 605 °C, (f) 880 °C, and (g) 965 °C, showing the improvement of the consolidation, surface porosity, and relative density by increasing milling time and annealing temperature.

**Fig. 13:**
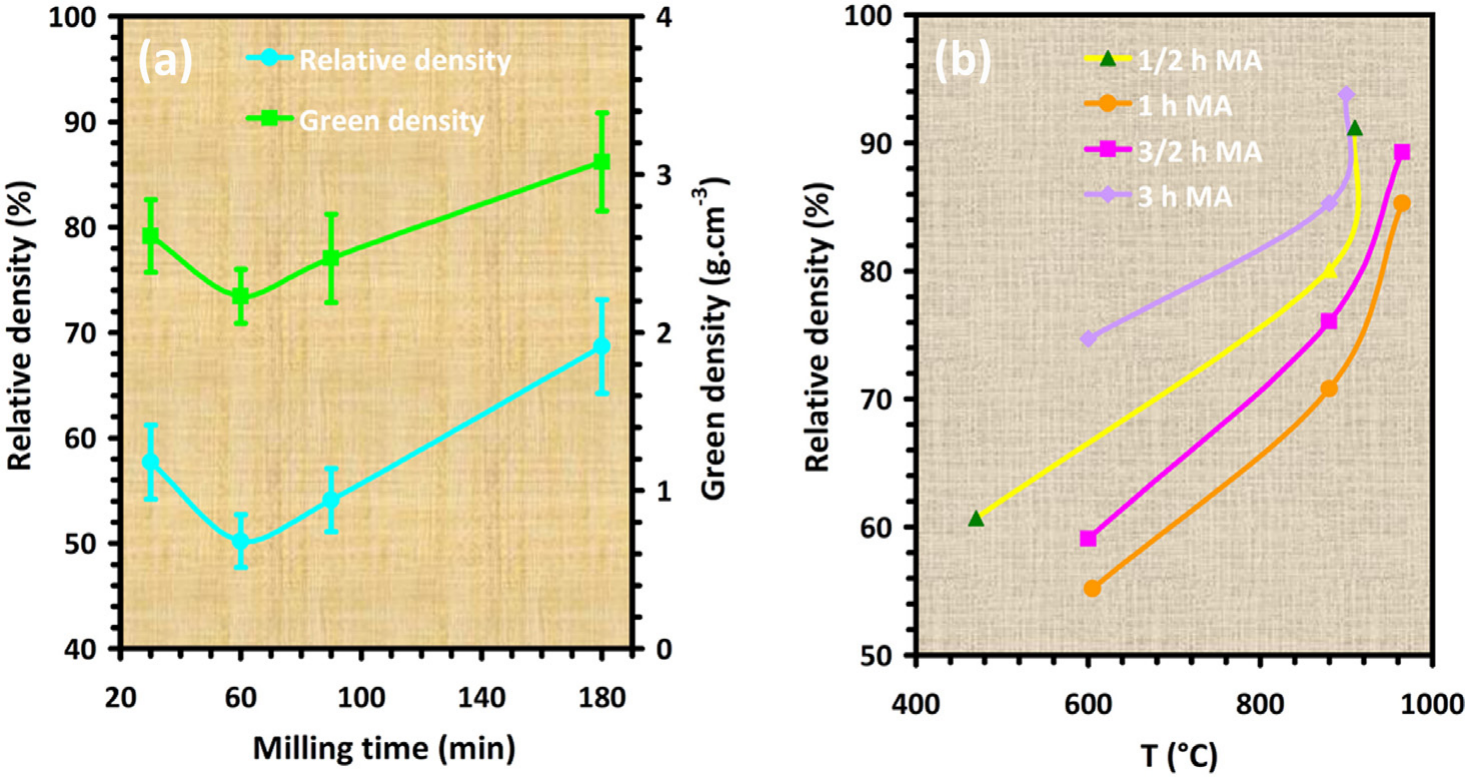
(a) Relative density and green density of consolidated as-milled AlCuFeB alloy powders, and (b) relative density of consolidated as-annealed AlCuFeB alloy powders at various temperatures. The Figures clearly show the enhancement of the relative density by increasing milling time and annealing temperature (For interpretation of the references to color in this figure legend, the reader is referred to the web version of this article.).

**Fig. 14:**
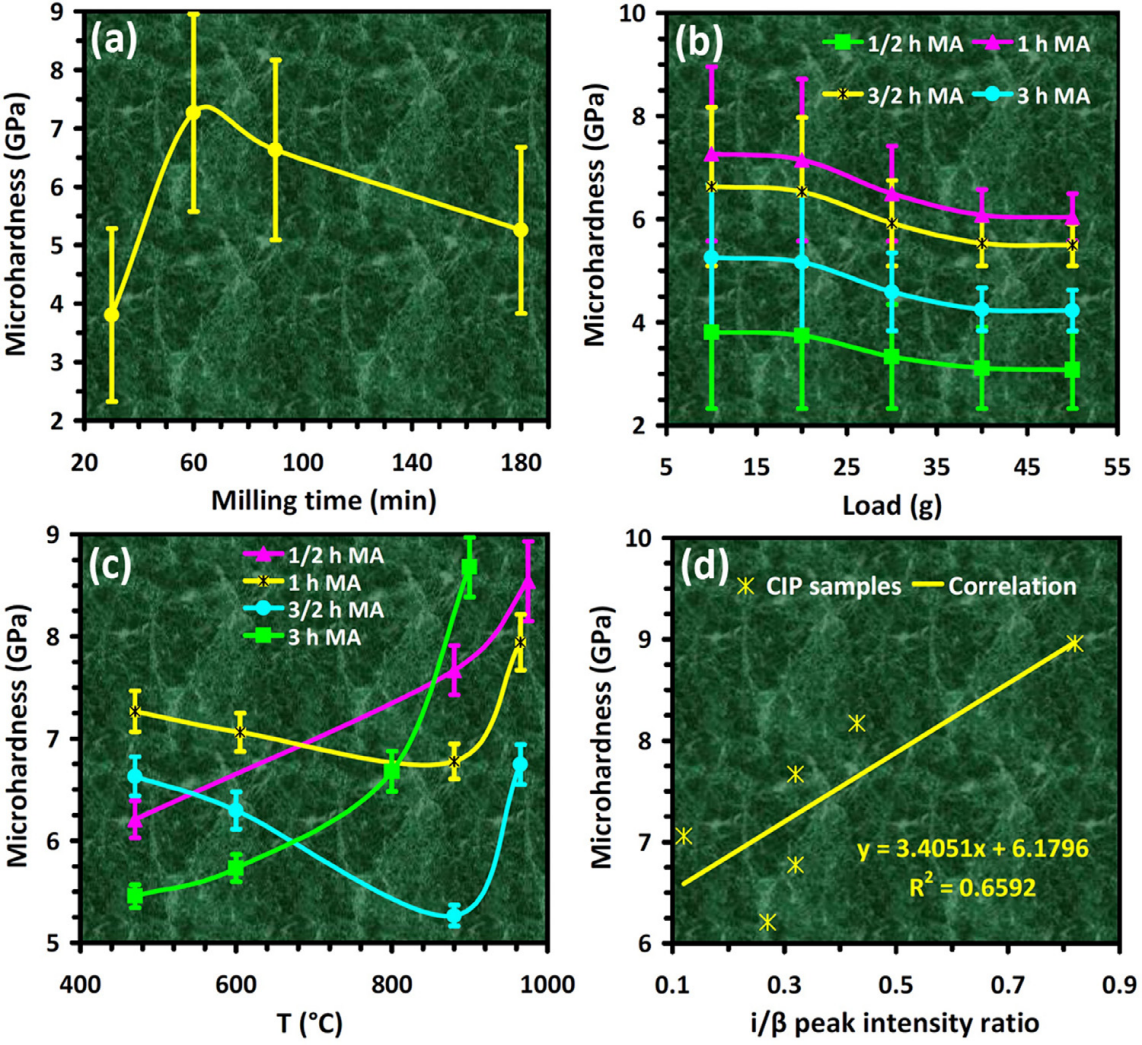
Characteristics of the AlCuFeB bulk CIP samples: (a) microhardness vs. milling time; (b) microhardness vs. indentation load; (c) microhardness vs. annealing temperature of the as-milled powders; (d) microhardness vs. i/β peak intensity ratio.

**Fig. 15:**
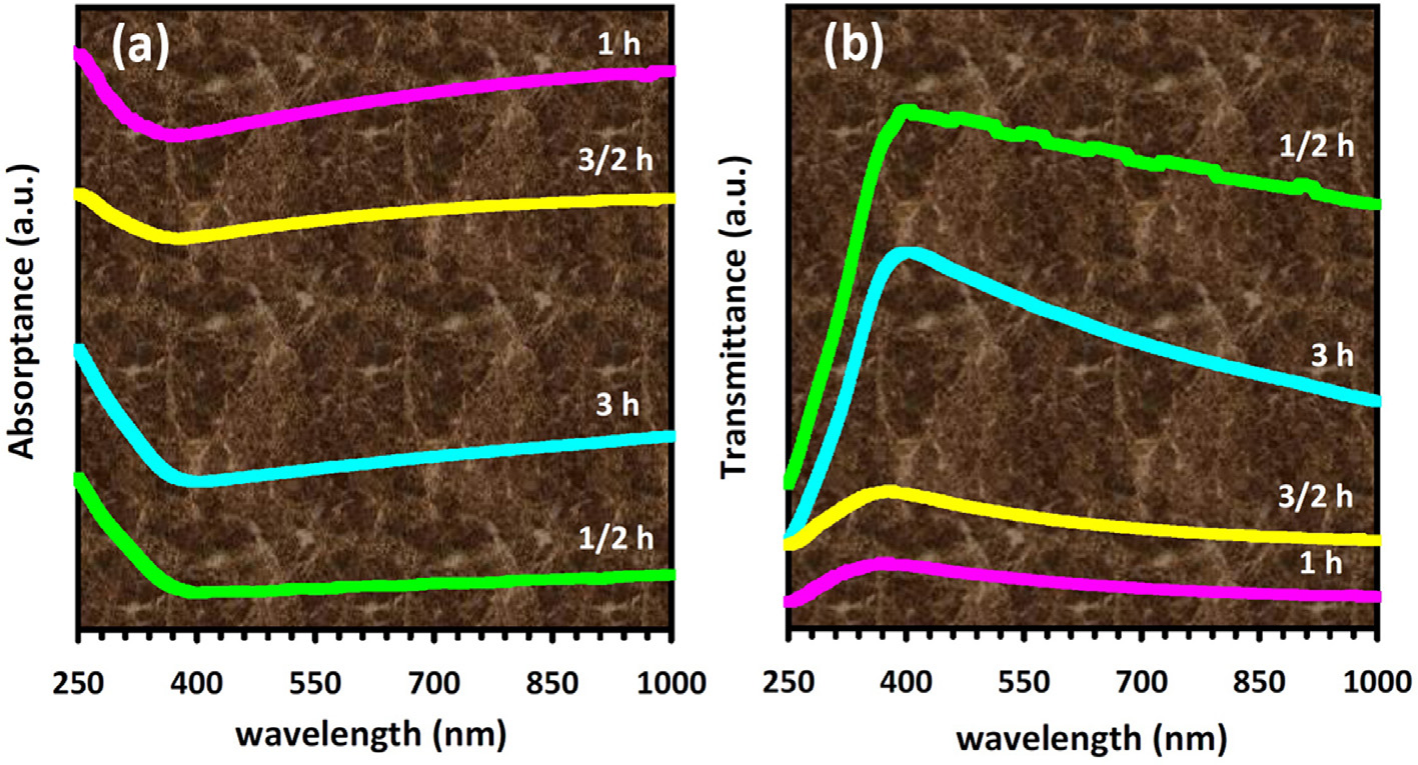
Optical properties of consolidated AlCuFeB as-milled samples: (a) absorptance spectra; (b) transmittance spectra. It shows the increasing and decreasing of the absorptance and transmittance in the wavelength range of 250–1000 nm, respectively.

The data presented here is on the evolution of the properties of the quasicrystalline phases [7]:

Mechanical alloying proved to be a simple way to synthesize intermetallic alloys.The dataset shows the influence of milling time on the quasicrystalline and other crystalline phases properties that form during ball milling process. Fig. 1 demonstrates the structural evolutions and phase composition of the as-prepared Al_59_Cu_25.5_Fe_12.5_B_3_ elemental powder blend obtained by mechanical alloying process for 1/2 to 3 h period. It should be noted that the ordered face-centered icosahedral (FCI) Al_59_Cu_25.5_Fe_12.5_B_3_ quasicrystal was obtained after 1 h of the MA process time. Table 1 represents the crystallographic data of the QC and β phases in the Al-Cu-Fe-B system after the 1 h milling process time [7]. The QC/crystallite size and microstrain were calculated by the Williamson–Hall method and optimized by the Rietveld refinement method, which is based on the fitting process of the corresponding XRD patterns. Table 2 reveals the evolution of the QC/β size and microstrain and β lattice parameter as a function of the milling process time [7]. It should be noted that the nature of the AlCuFeB QC phase, which is formed by short-time synthesis, is highly ordered, based on the XRD patterns. Fig. 2 illustrates the DTA and TGA curves of the un-milled and mechanically milled Al_59_Cu_25.5_Fe_12.5_B_3_ powders for 1/2 to 3 h. There are a series of exothermic and endothermic peaks in the temperature range of 400 to 1100 °C signifying the incidence of several solid-state transformations, including crystalline and QC evolutions during the annealing of the ball-milled powders. Fig. 3 shows the XRD patterns of the as-milled Al_59_Cu_25.5_Fe_12.5_B_3_ specimens after annealing at preferred temperatures in which the Phase transformations were readily observed. As shown in Fig. 3(b), the post-heat treatment of the Al59Cu25.5Fe12.5B3 alloy powder at 605 °C explained the notable reduction of the QC i-phase volume, and the β-phase was perceived to increase alternatively. The DTA curve of 1 h milled powder exhibits an endothermic at about 880 °C, which can be due to the depletion of AlCuFeB elements in various areas of the grain particles. Therefore, the QC i-phase completely transforms into the β intermetallic compound with increasing the annealing temperature to 880 °C, Fig. 3(b). Figs. 4–6 and 8–10 exhibit the secondary electron (SE) and backscattered electron (BSE) FESEM micrographs and the corresponding point and map EDS analyses for un-milled and as-milled Al_59_Cu_25.5_Fe_12.5_B_3_ powders. As shown in Fig. 4, the size and morphology (a), microstructure (b), elemental distribution (c), and chemical composition (d) of raw elements in un-milled Al_59_Cu_25.5_Fe_12.5_B_3_ powder observed. Fig. 5(a), the morphology of the powder particles is featureless. Moreover, some particles reveal faceted morphology, and the others are agglomerates of minute crystals. Fig. 5(b) demonstrates the presence of a mixture of the θ and λ phases besides the remaining primary elements. EDS mapping corresponded to Fig. 5(b) clearly shows a moderately uniform elemental distribution of Cu, Fe, and B alongside Al particles. Furthermore, Fig. 10(a) exhibits the chemical composition of the 1/2 h milled powder accomplished by the point EDS method that confirms the EDS mapping results. Fig. 6(a) shows the presence of rough particle morphology and inhomogeneous and disorder matted shape distribution of the Al_59_Cu_25.5_Fe_12.5_B_3_ powder particles after milling of 1 h. The microstructure of the QC i-phase and β-phase is shown in Fig. 6(b), which indicates a tangled mixture of agglomerated particles of their small crystals. The elemental distribution maps obtained for Al, Cu, Fe, and B exposes a relatively homogeneous distribution of Cu, Fe, and B elements dispersed within the Al agglomerates. Even more, the EDS maps corresponded to Fig. 6(b) show the localization of the Fe element in some areas. Additionally, the point EDS analysis of 1 h milled powder related to Fig. 6(b) interprets the presence and chemical composition of the Al, Cu, and Fe elements related to the β and QC phases, Fig. 10(b). Moreover, as shown in Fig. 7**,** the SAD patterns of the QC i-phase reveal the presence of two-fold, three-fold, and five-fold symmetries, which confirm the formation of the QC i-phase in the Al_59_Cu_25.5_Fe_12.5_B_3_ alloy system. The morphological features of 3/2 h milled powder are apparent in the SE-FESEM image that illustrates in Fig. 8(a). The morphology of the as-milled product was somewhat modified with increasing the milling process time to 3/2 h, as shown in Fig. 8(a). Although the particle size distribution has been homogenized much better, the morphology of several particles is still featureless, and particle agglomerates exist. It can be assumed that each particle in the agglomerates is a mixture of β and QC phases, Fig. 8(b). The EDS maps of the 3/2 h milled powder distribution directly reveal the uniform and homogenous distribution of Cu, Fe, and B within the Al particles. Furthermore, the corresponding point EDS has been shown in Fig. 10(c) in which illustrates the chemical composition of the chosen particle. Fig. 9(a) represents a relatively uniform and regular morphology of the Al_59_Cu_25.5_Fe_12.5_B_3_ powder particles following the 3 h BM process, which signifies the presence of only a few particle agglomerations. Fig. 9(b) shows the microstructure of 3 h milled powder particles containing only β phases. Through mapping the X-ray intensity of different chemical elements, their distribution could be associated with the β-phase microstructure. Moreover, the presence of the Al, Cu, and Fe can also be seen in Fig. 10(d) that it describes the chemical composition of the β-phase. Furthermore, Table 3 reveals the analytical data of the as-milled Al_59_Cu_25.5_Fe_12.5_B_3_ powder composition acquired by the ICP-AES examination, which shows a little variation within composition during the BM process [7]. The particle size distributions of the AlCuFeB alloy powders for various milling times are shown in Fig. 11. Fig. 12 demonstrates the surface OM images of the consolidated as-milled powders (1/2, 1, 3/2, and 3 h). Fig. 12 shows the surface OM images of the consolidated as-annealed powders subjected to 1 h MA as one of the consolidated heat-treated powders. Fig. 13 points out the green density and relative density of consolidated as-milled and as-annealed powders, which can readily analyze the density of all samples and observe their similarity. Fig. 14 shows the various characteristics of the as-milled and as-annealed bulk CIP specimens that have been represented as a function of the process parameters or inter-related properties. Table 4 reveals the *ρ* values of the as-milled and as-annealed consolidated samples [7]. As shown in Table 4, it seems that the *ρ* value of the annealed samples rises by increasing the volume fraction of the QC i-phase, which indicates that the effect of grain coarsening is almost negligible. Fig. 15 shows the optical absorptance and transmittance of the as-milled consolidated samples. It is well-known that the MA procedure notably effects on the microstructure of the powders, and subsequently on the optical properties of the pre-milled solid materials, which can be observed in Fig. 15 [7].

**Table 1:** Crystallographic propertiesof the QC and β structures in the AlCuFeB alloy system after the 1 h milling process time.

2θ°	d-spacing (Å)	Relative intensity (%)	FWHM	Phase/Ideal formula/Structure	Space group	Index
23.64	3.7683	25.17	0.6298	QC/Al_59_Cu_25.5_Fe_12.5_B_3_/Icosahedral	–	(222,000)
26.03	3.4233	22.69	0.0200	QC/Al_59_Cu_25.5_Fe_12.5_B_3_/Icosahedral	–	(311,111)
27.25	3.2722	24.28	0.1805	QC/Al_59_Cu_25.5_Fe_12.5_B_3_/Icosahedral	–	(2220–20)
30.37	2.9437	20.27	0.9446	β/Al_5_(Cu,Fe)_5_, AlFe(Cu)/Cubic	$\text{Pm}\bar{3}m$	(100)
42.87	2.1128	81.62	0.3149	QC/Al_59_Cu_25.5_Fe_12.5_B_3_/Icosahedral	–	(422,222)
3.56	2.0778	100.00	0.1181	β/Al_5_(Cu,Fe)_5_, AlFe(Cu)/Cubic	$\text{Pm}\bar{3}m$	(110)
45.05	2.0103	71.17	0.3840	QC/Al_59_Cu_25.5_Fe_12.5_B_3_/Icosahedral	–	(244,020)
63.86	1.4606	17.90	0.3115	β/Al_5_(Cu,Fe)_5_, AlFe(Cu)/Cubic	$\text{Pm}\bar{3}m$	(200)

**Table 2:** QC/β size and lattice microstrain beside the β lattice parameter as a function of the milling process time.

Milling time (h)	QC size (nm)	β size (nm)	QC strain (%)	β strain (%)	β lattice parameter (Å)
1	60.68	54.10	0.50	0.53	2.9346
3/2	52.78	42.23	0.64	0.69	2.9360
3	–	14.26	–	1.37	2.9362

**Table 3:** ICP-AES analysis of the as-milled Al_59_Cu_25.5_Fe_12.5_B_3_ alloy powder composition after various milling times.

Powder composition (at.%)	Al	CU	Fe	B
Un-milled	59	25.5	12.5	3
1/2 h ball-milled	58.8	25.5	12.7	3
1 h ball-milled	58.8	25.4	12.8	3
3/2 h ball-milled	58.7	25.4	13	2.9
3 h ball-milled	58.5	25.4	13.3	2.8

**Table 4:** Electrical resistivity (*ρ*) values of the mechanically alloyed and annealed AlCuFeB consolidated samples at room temperature.

Milling time (h)	Annealing temperature ( °C)	*ρ* (μΩ.cm)
1/2	–	132
	470	344
	880	430
	975	261
1	–	553
	605	410
	880	292
	965	450
3/2	–	511
	600	422
	880	282
	965	459
3	–	249
	900	243

## Conclusions

Thermodynamically stable quasicrystals are reasonably distinct from their crystalline counterparts. This phenomenon is due to their physicomechanical properties, such as electronic, optical, and mechanical properties. In this study, the Al_59_Cu_25.5_Fe_12.5_B_3_ nanoquasicrystalline alloy and related crystalline phases were synthesized through mechanical alloying using a high-energy ball milling and consolidated by a cold isostatic pressing apparatus. This paper focuses on the synthesis, structural and microstructural evolutions, and thermal stability of the Al_59_Cu_25.5_Fe_12.5_B_3_ nanoquasicrystalline alloys. Moreover, their microhardness besides electrical and optical properties was examined for solar selective absorber applications. Based on the results earlier reported, the conclusions can be as followed [7]:

• The nanostructured Al_59_Cu_25.5_Fe_12.5_B_3_ stable quasicrystalline phase was synthesized by short-time milling procedure in 1 h. Furthermore, the nanocrystalline B-Al(Cu,Fe) solid-solution was also formed. However, the single quasicrystalline phase could not be obtained even after the annealing treatment.
• The quasicrystalline size was calculated by the Williamson–Hall method and optimized by the Rietveld refinement procedure. It was found that the size is varied between 61 and 53 nm by increasing milling time up to 3/2 h. The crystallite size and lattice microstrain level of the β phase were adjacent to the saturation values around 14.26 nm and 1.37% following 3 h of milling time, respectively.
• The Vickers microhardness of the AlCuFeB bulk CIP samples was measured as a function of milling time, indentation load, annealing temperature of the as-milled powders, and i/β peak intensity ratio. It was diagnosed that the presence of the icosahedral phase notably affects the microhardness values.
• The electrical resistivity value of the mechanically alloyed and heat-treated AlCuFeB samples prepared through the CIP technique was estimated at room temperature. It was found that the highest electrical resistivity value of the consolidated samples belongs to 1 h ball-milled Al_59_Cu_25.5_Fe_12.5_B_3_ powders. Accordingly, it reaches a value of 553 μΩ.cm, which principally could be attributed to the presence of the quasicrystalline phase.
• The spectral analyses of absorptance and transmittance on the consolidated as-milled samples were carried out in the ultraviolet, visible, and near-infrared regions. It was turned out that the presence of the Al_59_Cu_25.5_Fe_12.5_B_3_ quasicrystalline alloy could improve the sunlight absorptance probably better than TiO_2_ thin films. Accordingly, the 1 h ball-milled sample has the highest absorptance and the lowest transmittance. This function could be related to the highest amount of the quasicrystalline phase at this stage of the BM process.

## Declaration of Competing Interest

The Authors confirm that there are no conflicts of interest..
